# Structural Interplays in the Flexible N-Terminus and Scaffolding Domain of Human Membrane Protein Caveolin 3

**DOI:** 10.3390/membranes11020082

**Published:** 2021-01-22

**Authors:** Hae-Jun Park, Jinhwa Jang, Kyung-Suk Ryu, Jinhyuk Lee, Sung-Hee Lee, Hyung-Sik Won, Eun-Hee Kim, Min-Duk Seo, Ji-Hun Kim

**Affiliations:** 1 College of Pharmacy, Chungbuk National University, Cheongju, Chungbuk 28160, Korea; gowns0419@chungbuk.ac.kr (H.-J.P.); zmdidi@naver.com (J.J.); suzukaze@naver.com (S.-H.L.); 2 Research Center for Bioconvergence Analysis, Korea Basic Science Institute, 162 Yeongudanji-Ro, Ochang-Eup, Cheongju, Chungbuk 28119, Korea; ksryu@kbsi.re.kr (K.-S.R.); keh@kbsi.re.kr (E.-H.K.); 3 Genome Editing Research Center, Korea Research Institute of Bioscience and Biotechnology (KRIBB), Gwahak-ro, Yuseong-gu, Daejeon 34141, Korea; jinhyuk@kribb.re.kr; 4 Department of Bioinformatics, KRIBB School of Bioscience, University of Science and Technology (UST), 217 Gajung-ro, Yuseong-gu, Daejeon 34113, Korea; 5 Department of Biotechnology, College of Biomedical and Health Science, Konkuk University, Chungju, Chungbuk 27478, Korea; wonhs@kku.ac.kr; 6 College of Pharmacy and Department of Molecular Science and Technology, Ajou University, Suwon, Gyeonggi 16499, Korea

**Keywords:** caveolin 3, nuclear magnetic resonance, paramagnetic relaxation enhancement, signature motif, oligomerization

## Abstract

Caveolins are critical for the formation of caveolae, which are small invaginations of the plasma membrane involved in a variety of biological processes. Caveolin 3 (Cav3), one of three caveolin isoforms, is an integral membrane protein mainly expressed in muscle tissues. Although various human diseases associated with Cav3 have been reported, structural characterization of Cav3 in the membrane has not been investigated in enough depth to understand the structure–function relationship. Here, using solution NMR, we characterized membrane association, structural communications, and molecular dynamics of the monomeric Cav3 in detergent micelle environment, particularly focused on the whole N-terminal part that is composed of the flexible N-terminus and the scaffolding domain. The results revealed a complicated structural interplay of the individual segments composing the whole N-terminal part, including the pH-dependent helical region, signature motif-like region, signature motif, and scaffolding domain. Collectively, the present study provides novel structural insights into the whole N-terminal part of Cav3 that plays important biological roles in cellular processes and diseases. In particular, given that several disease-related mutations are located at the whole N-terminal part of Cav3, the sophisticated communications in the whole N-terminal segments are likely to have relevance to the molecular basis of Cav3-related disease.

## 1. Introduction

Caveolae are small invaginations presented in the plasma membranes of a wide range of mammalian cell types, such as adipocytes, endothelial cells, fibroblasts, and smooth muscle cells [1,2]. Caveolae are enriched in cholesterol, glycosphingolipids, and lipid-anchored membrane proteins and play important roles in a wide variety of cellular processes, including endocytosis, cellular signaling, lipid metabolism, and mechanosensing [3,4,5,6,7,8,9]. The principal structural proteins called caveolins including Caveolin 1 (Cav1), Caveolin 2 (Cav2), and Caveolin 3 (Cav3) drive the formation of caveolae on the plasma membrane [10,11,12]. Cav1, the most extensively characterized one so far, is ubiquitously expressed in various tissues, including adipocytes, endothelia, and type-1 pneumocytes, together with Cav2. In contrast, Cav3 is mainly expressed in the skeletal and smooth muscle tissue [13]. Some mutations on Cav3 are associated with a variety of neuromuscular and cardiac disorders, including limb-girdle muscular dystrophy 1C (LGMD 1C), HyperCKemia, distal myopathy, and inherited rippling muscle disease-2 (RMD2) [14,15,16,17,18,19,20,21,22]. In particular, more than 70% of RMD2-related mutations are located at the flexible N-terminus and scaffolding domains (Figure S1). However, the relationship between the structure and function of Cav3 in the membrane has not been revealed. Thus, structural studies on Cav3 in the membrane are essential to understand various human diseases linked to Cav3 mutations.

**Figure 1:**
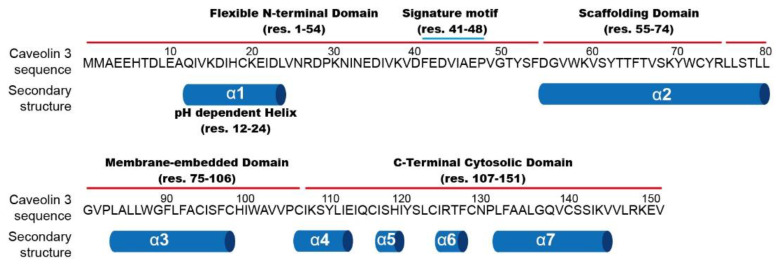
Amino acid sequence and secondary structure of Caveolin3. Structural domains depicted with red line on the amino acid sequence include the flexible N-terminal domain, the scaffolding domain, the membrane-embedded domain, and the C-terminal cytosolin domain. Blue line indicates the signature motif.

Although Cav1 is the most frequently characterized caveolin [23,24,25], Cav1 and Cav3 are highly homologous (Cav3 is 65% identical and 85% similar to Cav1), which indicates that Cav1 and Cav3 are likely to adopt similar topology and structure. Caveolins have an unusual structure where both N- and C-termini face the cytoplasm. It is believed that the helix-break-helix topology of the central hydrophobic segment embedded in the membrane effectively splits the molecule into two cytoplasmic domains. As shown in Figure 1, Cav3 is composed of four structural domains: a flexible N-terminal domain ((NTD) residues 1–54), a scaffolding domain ((CSD) 55–74), a membrane-embedded domain (75–106), and a C-terminal cytosolic domain ((CTD) 107–151) [26]. The flexible NTD contains a pH-sensitive helix region and a signature motif (residues 41–48 (FEDVIAEP)), highly conserved among the caveolin family [27]. The CSD is a critical domain for membrane association and cholesterol binding, and as well as homo- and hetero-oligomerization [28,29,30,31]. The region containing both the flexible N-terminus and the scaffolding domain is termed the whole N-terminal part. The membrane-embedded domain consists of a U-shaped helix-break-helix motif that penetrates deeply into membrane bilayers [32,33]. Lastly, the cytosolic CTD of Cav3 is composed of four α-helices associated with the membrane surface. Although the CTD also contains three palmitoylations on three cysteine residues, the previous investigation using in vitro lipidation suggested that these palmitoylations have a modest effect on the local structure of the CTD [34].

Although various structural characteristics of caveolin have been identified, some unanswered questions regarding the precise mechanism for the formation of an oligomer and the function of the signature motif remain. The formation of caveolae critically depends on the oligomerization of caveolin proteins. Cav1 and Cav3 can each assembles into a low-molecular-weight oligomer, which serves as the most basic unit forming the caveolae [30,35]. Those basic oligomers then undergo the next stage of oligomerization. However, the identity of the caveolin region mediating each oligomerization is the subject of debate. Initially, it was believed that the formation of low-molecular-weight oligomers was mediated by CSD, while high-molecular-weight oligomers were formed through a side-by-side packing of CTD [30,35]. However, a recent electron microscope (EM) investigation of Cav3 showed a different assembly of the basic oligomer unit, which was critically mediated by the CTD instead of the CSD [36]. Next, functional role of the signature motif that is highly conserved in caveolin families is a subject to be elucidated. Although this signature motif is also known to be involved in oligomerization of caveolins, it is not clear how this motif region contributes to the oligomerization. Based on the EM structure of C-terminal truncated Cav1 and Cav3, it was proposed that the acidic patch around the signature motif binds to the amino acids that spiral up the helix of the CSD, causing the N-terminal tail to wrap around the helix [37]. However, since then, there has been no evidence clarifying the exact function of the flexible NTD, including the signature motif. In summary, caveolin oligomers play critical roles in the formation of caveolae on the plasma membrane and structural properties of caveolin monomers can provide the fundamental driving force for the formation of oligomers. Our previous NMR investigations on monomeric Cav3 revealed secondary structure rearrangement in the flexible NTD in 1-palmitoyl-2-hydroxy-sn-glycero-3-phosphoglycerol (LPPG) micelles as an optimal membrane-mimetic environment [27]. In this study, we focused on the structural characterization of the flexible NTD and the CSD based on a variety of NMR experiments including paramagnetic relaxation enhancement (PRE), chemical shift perturbation (CSP), and ^15^N Carr–Purcell–Meiboom–Gill (CPMG) relaxation dispersion analysis in LPPG micelles.

## 2. Materials and Methods

### 2.1. Protein Expression and Purification

Constructs used in this work contained a Cav3 mutant where six of the native Cys sites were replaced with Ala or Ser (C19S, C72A, C94A, C98A, C124S, and C140S). The fifth, sixth, and eighth Cys sites (C106, C116, and C129) from the N-terminus were mutated to Phe or lipidated. Mutant variants were generated via site-directed mutagenesis using the QuikChange site-directed mutagenesis kit (Agilent, Santa Clara, CA, USA). Expression, purification, incorporation of LPPG membrane mimetic, and lipidation at Cys sites were performed as previously described [27,34].

### 2.2. DSA-Induced Paramagnetic Peak Reduction to Probe Membrane Topology

The 2D ^1^H-^15^N-HSQC spectrum of lipidated Cav3 was collected in the presence of lipophilic 16-doxylstearic acid (16-DSA) on a BRUKER Avance 900 MHz at 45 °C. The 16-DSA stock was first prepared by dissolving in methanol at a final concentration of 2.5 mg/mL. The proper amount of the 16-DSA stock to make a final concentration of 4 mol% in the micelle phase was then dispensed into an Eppendorf tube, followed by evaporation of the methanol. The micellar Cav3 sample was then transferred into the Eppendorf tube to solubilize 16-DSA and the pH was adjusted to 7.2. NMR samples contained 180 µM 15N-lipidated Cav3 in a buffer containing 100 mM imidazole, 5% (*w*/*v*) LPPG, 2 mM EDTA, 10% D2O, and the indicated concentration of DSA. The ratios of peak intensities in the spectrum of a DSA-containing sample to those of the corresponding peaks of a diamagnetic sample were measured. All spectra measured in the present work were processed using NMRPipe [38] and analyzed using Sparky [39] or NMRView [40].

### 2.3. PRE Experiments and Structural Analysis

For PRE measurements, proteins were prepared in which natively palmitoylated cysteines were mutated to Phe and other cysteines were mutated to Ser or Ala as described above. The single-Cys mutant was purified into LPPG and spin-labeled using an established method. Briefly, each single-Cys mutant was concentrated to 0.3 mM, and the pH was adjusted to 7.2 followed by the addition of 2.5 mM DTT to ensure Cys thiol reduction. The reduced single-Cys mutant was then spin-labeled by adding excess thiol-reactive probe S-(1-oxyl-2,2,5,5-tetramethyl-3-pyrroline-3-methylmethanethiosulfonate) ((MTSL) Toronto Research Chemicals Inc., Ontario). A 25-fold excess of MTSL was added relative to ~0.3 mM mutant Cav3 in the buffer (100 mM imidazole, 2 mM EDTA, 2.5 mM DTT, and 0.2% LPPG (pH 7.2)). The reactant was incubated overnight, followed by buffer exchange with 50 mM sodium phosphate buffer (pH 7.2) to remove imidazole in preparation for his-tag affinity chromatography. The solution was then mixed for 1 h with Ni-nitrilotriacetic acid resin and the resin was washed with 30 × 1 column volumes of 50 mM phosphate and 0.2% LPPG (pH 7.8) to remove excess MTSL and MTSL-modified DTT. The protein was eluted in the same 0.2% LPPG micelle with 250 mM imidazole at pH 7.8. The sample was then immediately transferred into the 100 mM imidazole (pH 7.2) buffer containing 2 mM EDTA.

The 2D ^1^H-^15^N HSQC spectra of solutions containing spin-labeled single-Cys mutant in the absence or presence of the reducing agent sodium ascorbate were measured using a BRUKER Avance 800 MHz spectrometer operating at 45 °C. The intensities of cross-peaks in the ¹H-^15^N HSQC spectra of both the spin-labeled mutant in its oxidized state and the reduced mutant with an excess of sodium ascorbate were measured and their ratio was calculated. A fully extended model structure was generated via a short minimization simulation using CHARMM software [41] to explore the conformational preferences. The peak intensity ratio between the paramagnetic (*I_P_*) and the diamagnetic (*I_D_*) states of the random coil model was calculated using the following formula [42]:

(1) $$\frac{I_{P}}{I_{D}}=\frac{R_{2D}\exp\left(-R_{2P}t\right)}{R_{2D}+R_{2P}}$$

where *R*_2*D*_ is the transverse relaxation rate in the diamagnetic state, which is set to 7.5 s^−1^, the approximate average *R*_2_ is obtained using NMR for N-terminus of Cav3, and t is the duration of the delays in HSQC pulse sequence, which is set to 4 ms. *R*_2*D*_ is the paramagnetic contribution to the transverse relaxation rate, which is calculated as follow [42]:

(2) $$R_{2P}=\frac{K}{r^{6}}\left(4\tau_{C}+\frac{3\tau_{C}}{1+\omega_{H}^{2}\tau_{C}^{2}}\right)$$

where r is the distance between each residue and the spin-label (residue numbers: 38, 53, 61, and 72), *K* is constant (1.23 × 10^−32^ cm^6^s^−2^), *ω_H_* is the Larmor frequency of a proton, and *τ*_C_ is the effective correlation time for the used protein (estimated at 5.5 ns based on NMR relaxation measurements taken previously).

### 2.4. Protein Dynamics’ Analysis Using Nitrogen CMPG Dispersion Experiments

Nitrogen relaxation experiment was conducted using ^15^N-labeled Cav3. Single quantum ^15^N CPMG was carried out using relaxation compensated TROSY pulse sequences with constant time delays of 40 ms at 600 MHz. The time spacing between the 180° pulse centers was equal to 2*τ_cp_*, which was expressed as a frequency (*ν_CPMG_*) equal to 1/(4*τ_cp_*). For the first sample, 2D data sets were acquired for different *ν_CPMG_* values (25, 50, 75, 100, 150, 200, 300, 400, 550 Hz) at *B*_0_ = 14.1 (at 45 °C). Reference spectra were acquired with no constant time delay.

*R*_2*eff*_ was calculated using the following formula:

(3) $$R_{2eff}\left(v_{CPMG}\right)=-\frac{1}{Trelax}\cdot \ln\left(\frac{I\left(v_{CPMG}\right)}{I_{0}}\right)$$

where *I*(*ν_CPMG_*) is the intensity for a given *ν_CPMG_* value, *I*_0_ is the intensity of the reference spectrum, and *T* is the constant time delay. CPMG nitrogen dispersion experiments were performed using a 600-MHz BRUKER spectrometer. The transverse relaxation rate was fitted using the Carver-Richards expression [43]:

(4a) $$R_{2}\left(v_{CPMG}\right)=\frac{1}{2}\left(R_{2A}^{0}+R_{2B}^{0}+k_{ex}-2v_{CPMG}cosh^{-1}\left(D_{+}\cosh\left(\eta_{+}\right)-D_{-}\cosh\left(\eta_{-}\right)\right)\right)$$

(4b) $$D_{\pm }\;=\;\frac{1}{2}\left(\frac{2\Delta w_{AB}^{2}}{\sqrt{\psi^{2}+\;\xi^{2}}}\pm 1\right)$$

(4c) $$\eta_{\pm }=\;\frac{\sqrt{2}}{4v_{CPMG}}\sqrt{\pm \psi +\sqrt{\psi^{2}+\xi^{2}}}$$

(4d) $$\psi ={\left(\;R_{2A}^{0}-R_{2B}^{0}+p_{B}k_{ex}-p_{A}k_{ex}\right)}^{2}-△w_{AB}^{2}+4p_{A}p_{B}k_{ex}^{2}$$

(4e) $$\xi =2\Delta \omega_{AB}\left(R_{2A}^{0}-R_{2B}^{0}+p_{B}k_{ex}-p_{A}k_{ex}\right)$$

where *R*_2*A*_^0^ and *R*_2*B*_^0^ are the intrinsic transverse relaxation rates in states *A* and *B*, respectively, and *k_ex_* is the exchange rate.

## 3. Results and Discussion

### 3.1. Membrane Topology Probe Using DSA-Induced Paramagnetic Peak Reduction

**Figure 2:**
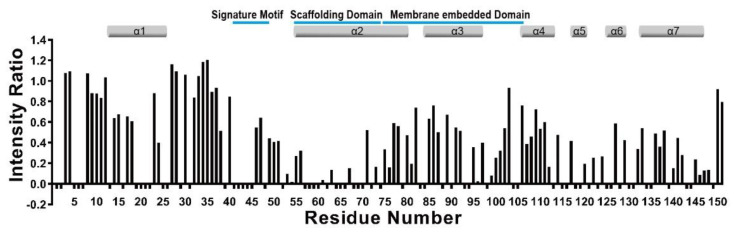
The intensity of backbone amide peaks in the presence of 4 mol% lipophilic paramagnet, 16-DSA, relative to that measured in the absence of paramagnetic agent is shown for each residue. Negative bars represent residues for which the ratio could not be accurately determined.

To date, the precise function of the flexible NTD of Cav3, including the signature motif, has been unclear. The EM structure of the C-terminus-truncated Cav3 suggested that the N-terminal tail wraps around the helix; however, since then, a detailed interplay of the flexible NTD in the full-length protein has not been elucidated with atomic resolution. To figure out the structural characteristics, the topological feature of mutant Cav3, termed the lipidated 5F68C-Cav3, in which the fifth Cys was replaced to Phe, the sixth and eighth Cys residues were lipidated via alkyl group conjugation, and other Cys residues were replaced to Ala or Ser, was first investigated using 16-DSA incorporated into the LPPG micelle at pH 7.2 (Figure 2). As expected, all residues in the CSD were buried in the membrane. However, interestingly, peak broadening started from residue 35 instead of residue 55, which is first residue of the CSD, indicating that the signature motif was embedded into the membrane, as shown in Figure 2. There are two hypotheses to explain why this region was embedded in the membrane. The first is that the signature motif bound to the scaffolding domain, as shown in the EM structure, and the second is that this region embedded itself in the membrane environment.

### 3.2. Structural Analysis Using MTSL-Induced Paramagnetic Peak Reduction

**Figure 3:**
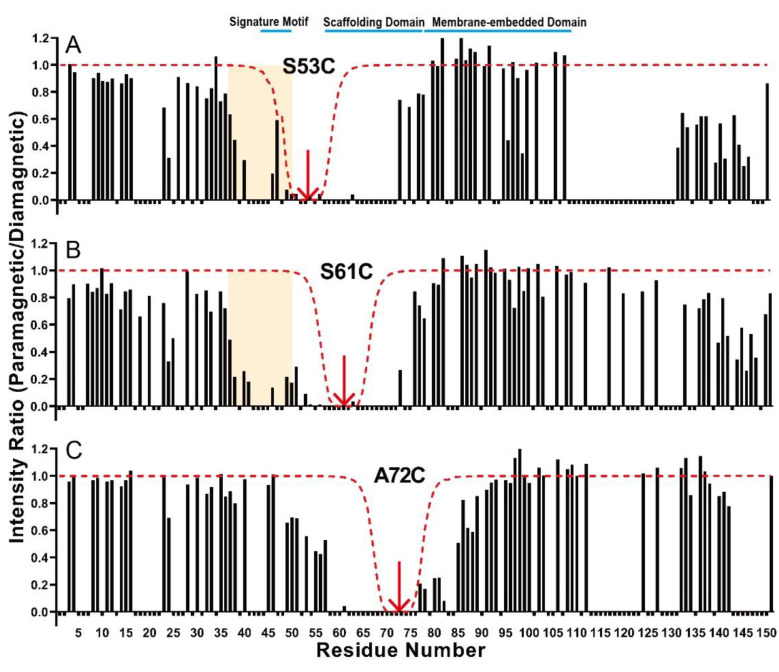
Intensity ratio plots for spin-labeled Cav3. The histograms represent the ratio of cross-peak intensities in the ^1^H-^15^N HSQC spectra of the oxidized and reduced states, as well as paramagnetic and diamagnetic states of the MTSL-labeled Cav3 at each S53C (**A**), S61C (**B**), and A72C (**C**) mutation. The yellow box indicates the decrease of the intensity ratio in the flexible NTD. The red, dotted line indicates the broadening, predicted using a simulated extended structure. Negative bar indicates residues that could not be assigned.

Although membrane topology analysis based on DSA-induced paramagnetic effect helps to understand the characteristics of the whole N-terminal part of Cav3, it is not sufficient to figure out the precise role. Therefore, site-specific PRE experiments were conducted to obtain more structural information. By attaching spin labels to cysteine sites in the Cav3 mutant, it is possible to estimate the physical distance between the spin-labeled site and other locations within the protein through intensity ratios of paramagnetic versus diamagnetic samples. Here, because the majority of peaks in the scaffolding domain were missing or overlapping in the 2D ^1^H-^15^N HSQC spectrum, PRE analysis through the spin labeling at the signature motif was not efficient to detect the interaction between the flexible NTD and the CSD. Therefore, MTSL was introduced at three sites, S53 residue located just before the CSD and S61 and C72 located in the CSD, to investigate the degree of spatial proximity between the CSD and the flexible NTD. In order to determine the degree of proximity from the spin-labeled site, the intensity ratios from experimental results were compared with the intensity ratios from the simulation curve using the fully extended structure. As shown in Figure 3, spin labels at S53 and S61 showed a similar intensity ratio where the intensity ratio rapidly decreased from residue 35, which corresponds to the result of DSA-induced PRE experiments. The decrease curves of intensity ratio in the flexible NTD were different from the simulation curve from the fully extended structure. In contrast, the intensity ratio around residue 35 was not affected by paramagnetic spin labels at C72. Compared to the simulation curve of the fully extended structure, a decrease in the intensity ratio of the flexible NTD was a relevant result, indicating a close distance between the N-terminus of the CSD and the signature motif. For the proximity between the CSD and the signature motif, negatively charged residues clustered in the signature motif (residues 41–48, FEDVIAEP) might play an important role in the direct interaction with positively charged residues in the CSD (residues K59, K69, and R74) via electrostatic interaction.

Interestingly, there was another region (residues 33–40 NEDIVKVD) that contained similar sequences to the signature motif, including a cluster of negatively charged residues located just before the signature motif. Hereinafter, this region is termed the signature motif-like region. They were also highly similar to the corresponding residues in Cav1 (those of Cav3 were 63% identical and 100% similar to those of Cav1). For these reasons, both clusters of negatively charged residues were considered to be involved in the association with the CSD.

### 3.3. Chemical Shift Comparison Caused by Mutations

**Figure 4:**
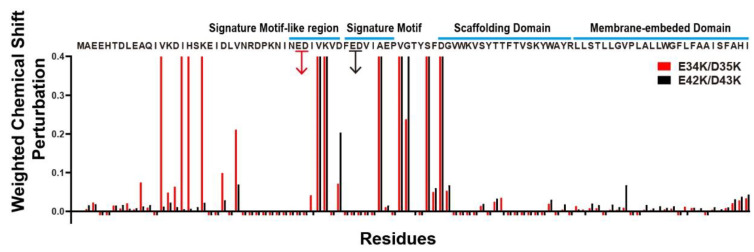
Chemical shift perturbations caused by E34K/D35K (red) and E42K/D43K (black). Bars that reach to the maximum y value denote residues, for which the peak shows a huge difference, including the chemical shift change (>0.4) or peak disappearance via mutation. Negative bars in the histogram indicate the residues that could not be assigned because of peak broadening and overlapping.

To investigate key residues for the proximity of the flexible NTD and the CSD, NMR experiments were performed to obtain CSPs caused by the mutation (Figure 4). Negatively charged residues (E34, D35, E42, and D43) that were predicted to contact with the scaffolding domain via electrostatic interactions were selected and mutated to the oppositely charged amino acid, Lys. Therefore, two mutated constructs, containing E34 and D34, and E42 and D43, were changed to Lys, making E34K/D35K and E42K/D43K, respectively. Chemical shifts of assigned residues in the CSD of mutants were expected to show big differences, as the mutated residues directly interfered with the strong electrostatic interaction between the flexible NTD and the CSD. Unfortunately, the mutation effects on the CSD could not be concluded because only a few peaks were observed in the CSD due to peak broadening and ambiguous peak assignments, as shown in Figure 4. Instead, Figure 4 shows both mutants E34K/D35K and E42K/D43K caused substantial CSPs in a broad range of the flexible NTD, which had not been reported before. It is important to note that the interaction of positively charged amino acids mutated from Asp and Glu with the negatively charged head of LPPG possibly resulted in distorted structural information. Nevertheless, as the two mutated amino acids were bound to the membrane, it would be difficult to make changes in a wide range of chemical shifts. Therefore, mutation analysis suggested that there are complicated structural interplays in the flexible NTD, which results in forming an unrevealed structure. We predicted that the signature motif and the signature motif-like region played important structural roles in the unrevealed N-terminus structure. In the same context, the unrevealed and complicated interactions may affect the proximity between the flexible NTD and the CSD, suggested via paramagnetic experiments.

Although a particular secondary structure was not found in the flexible NTD at pH 7.2 based on the previous NMR data, the present results indicated a broad range of interplays in the whole N-terminal part. To date, the structural characteristics of the whole N-terminal part have not been clearly identified, and some proposed characteristics remain controversial. For example, there are two conflicting hypothetical models for the membrane association of CSD, which have been reported: (1) the cytosol exposed model where the N-terminal region of CSD is exposed to the cytosol and (2) the membrane-buried model where the N-terminal region of CSD is buried into membrane. Our present results are more suitable for the membrane-buried model. In addition, there is a debate on whether the secondary structure of the N-terminal region of CSD is α-helix or β-strand. Hoop and his colleagues demonstrated that the N-terminal region of the CSD of Cav1 was composed of a β-strand, whereas our previous results showed α-helix in the corresponding region of Cav3.

These inconsistent structural characteristics are possibly caused by a limitation of the membrane mimetic system that does not reflect an accurate biological membrane. Especially, considering that caveolae are specialized lipid rafts that are dynamic ensembles containing sphingolipids and cholesterol in the membrane, membrane mimetics are expected to be quite different from a biological membrane. Although the exact mechanism for the interaction between cholesterol and caveolin has not yet been elucidated, there is no doubt that caveolin is related to cholesterol. Therefore, the presence of cholesterol is highly likely to affect the structural characteristics of membrane proteins in vitro. We also found chemical shift perturbations of residues located in the membrane-associated region upon adding cholesterol to Cav3 in the LPPG micelle system (Figure S2). The comparison between HSQC spectra with and without cholesterol showed that the residues located at the membrane-associated region, including the CSD, were mainly affected by adding cholesterol, which indicated that cholesterol was an influential factor in determining the structural characteristics of Cav3. The detailed study on the differences in the structural characteristics of Cav3 depending on whether membrane mimetics contain cholesterol will be conducted in the future.

As another factor, the characteristics of the LPPG, such as the charge and shape of the head group, can affect the structural features of the membrane protein. Previously, several detergents were screened for structural studies on Cav3 using NMR. It was confirmed that Cav3 incorporates into a charged detergent, such as dodecylphosphocholine (DPC), n-tetradecylphosphocholine (TDPC), lyso-myristoylphosphatidylglycerol (LMPG), and LPPG, via detergent exchange on the histidine affinity column, in the monomeric form [34]. However, Cav3 was not eluted when it was incorporated into n-Dodecyl β-D-maltoside (DDM), which might be caused by protein aggregation inside the column. In the same context, EM study on Cav3 showed the structure of a low-molecular-weight oligomer in the DDM micelle system. As a result, the structural characteristics of Cav3 vary depending on the detergent characteristics. In addition, our previous investigations demonstrated that pH also has an important influence on the residues between 12–24 in the flexible NTD where the pH-dependent conformational exchange between random coil and helix was detected [27].

Taken together, inconsistent structural characteristics of the CSD, differences in the oligomeric states of Cav3 depending on the detergent micelle as well as pH-dependent conformational changes in the flexible NTD, suggest that the whole N-terminal part is likely to possess a dynamic structure depending on the surrounding environment, which is possibly related to new functions that have not been previously reported.

### 3.4. Nitrogen Relaxation Dispersion Experiment

For the reasons described above, we aimed to check the structural dynamics. In particular, global motions, such as conformational changes and the folding process described here, are to fall in the microsecond to millisecond timescale. It is proposed that conformations of proteins pre-exist in different populations and the interactions happen through a conformational selection mechanism. CPMG relaxation dispersion NMR experiments were developed to investigate these structural motions and conformations. Peak-broadening analysis of the CPMG-relaxation dispersion spectra provide *R*_2eff_ values, implying conformational information at each different CPMG time delay.

**Figure 5:**
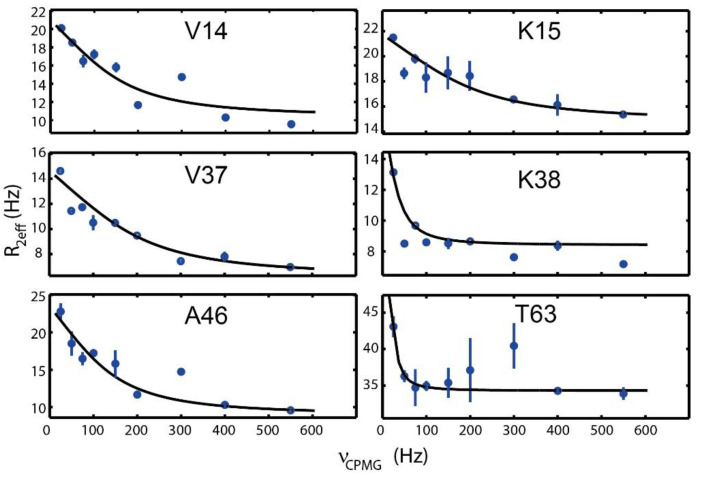
Nitrogen CPMG relaxation dispersion measurements for V14, K15, V37, K38, A46, and T63 recorded at 318 K. Calculated transverse relaxation rates (*R*_2eff_) plotted as a function of *ν*_CPMG_ = 1/(2*τ*), where τ is the delay between successive refocusing pulses. The size of the error bars is the standard deviation computed from duplicated experiments.

In the present work, we plotted calculated *R*_2eff_ against *ν*_CPMG_ measured under one condition (600 MHz and 318K) and investigated whether each peak underwent an exchange instead of calculating the exchange rate or population of conformations via multivariate data fitting. The detergent micelle is not a proper membrane mimetic system, considering that the flexibility of membrane proteins is compromised in the micelle membrane system [44]. Nevertheless, the selected residues V14, K15, V37, K38, A46, and T63 in the whole N-terminal part show dynamic behavior according to the R_2eff_ fitting, as shown in Figure 5. V14 and K15 belong to the region (residues 10–24) that have a pH-dependent conformational change between the helix and the random coil [27]. Although this region is composed of a random coil at a high pH, the transition to α-helix was predicted to exist in a minor population following a slow exchange mode. Based on the mutation experiments (Figure 4), this region was associated with the signature motif-like region and the signature motif, which implied that pH is one of the key factors regulating global interplays in the whole N-terminal part. V37 and K38, and A46, were located at the signature motif-like region and the signature motif, respectively, which suggested that these regions also underwent structural dynamics including intra-residue interaction and/or conformational changes in the microsecond to millisecond time scale. Since most residues belonging to the signature motif could not be assigned due to peak broadening, structural dynamics were not directly confirmed via nitrogen dispersion experiments. However, peak broadening of partial residues was largely due to exchange processes on the millisecond to microsecond time scales; therefore, missing peak of the signature motif could be another evidence to prove structural dynamics of this region. Our results also supported that the signature motif-like region and the signature motif contributed to the global dynamics of the whole N-terminal part in a millisecond to microsecond time scale. Thus, the unrevealed structure of the whole N-terminal part was expected to vary.

It is challenging to obtain structural information of the CSD through nitrogen dispersion experiments because membrane association of the CSD causes slow tumbling time and short T2 time, and then the signal is difficult to maintain during the pulse sequence. Accordingly, we could not obtain *R*_2eff_ values for several residues in the CSD. Nevertheless, T63 showed dynamic behavior, as shown in Figure 5. Dynamic behavior of the CSD could potentially be related to one or more of following factors: (1) structural transition between α-helix and β-strand, (2) the switch between being exposed to the cytosol and being embedded to the inner membrane, (3) interaction of the scaffolding domain with other intra-regions. The exact reason for the dynamic behavior of the CSD will be explored in further work. In summary, motional dynamics appearing in a wide range of the whole N-terminal part also support the global interplays among pH-dependent conformational region, signature motif-like region, signature motif, and scaffolding domain, suggested by the mutational studies described above. Furthermore, structural dynamics in the wide range of the whole N-terminal part provided a clue for understanding the mechanism of the disease-related mutation in this region that has not been reported previously.

## 4. Conclusions

Although Cav3 is associated with a variety of muscle diseases, the structural characteristics are unclear. To date, studies on structural characteristics of the flexible NTD have been neglected, and inconsistent characteristics have been reported for the CSD. In the present work, structural features and topology of Cav3, along with those of the membrane, were investigated using solution NMR. N-terminus of the CSD is close to the signature motif. Moreover, the signature motif-like region is close to both the pH-dependent transient α-helical regions in the flexible NTD and the signature motif. Residues belonging to those core regions, such as the pH-dependent transient α-helical region, the signature motif-like region, the signature motif, and the CSD, show structural dynamics in a millisecond to microsecond time scale. Taken together, each core region in the whole N-terminal part is expected to have complicated structural communications, which has not been reported elsewhere. In addition, the present work suggests that Cav3 has a dynamic structure, which may explain the inconsistent structural properties reported before, depending on the surrounding environment. These structural features are possibly relevant to muscle-related diseases. However, to clarify these suggestions, it is necessary to analyze the structural characteristics of Cav3 in various surrounding environments in further studies.

## Supplementary Materials

The following are available online at https://www.mdpi.com/2077-0375/11/2/82/s1. Figure S1: Disease-related amino acids of Cav3. HYPCK, HyperCKemia; RMD2, rippling muscle disease-2; LGMD1C, limb-girdle muscular dystrophy (LGMD) type 1C. Figure S2: HSQC spectra of Cav3 in LPPG micelle with (red)/without (black) cholesterol.
