# Supplementary material for: Structural Interplays in the Flexible N-Terminus and Scaffolding Domain of Human Membrane Protein Caveolin 3
Source: Membranes (Basel). 2021 Jan 22;11(2):82. doi: 10.3390/membranes11020082 (PMC7912387; doi:10.3390/membranes11020082)
Supplement: Supplementary file 1 [file membranes-11-00082-s001.pdf]

Article

Supplementary Materials: Structural Interplays in the Flexible N-Terminus and Scaffolding Domain of Human Membrane protein Caveolin 3

Hae-Jun Park [1,†], Jinhwa Jang [1,†], Kyung-Suk Ryu [2], Jinhyuk Lee [3,4], Sung-Hee Lee [1], Hyung-Sik Won [5], Eun-Hee Kim[2], Min-Duk Seo [6,*] and Ji-Hun Kim [1,*]

1 College of Pharmacy, Chungbuk National University, Cheongju, Chungbuk 28160, Republic of Korea; gowns0419@chungbuk.ac.kr (H.-J.P.); zmdidi@naver.com (J.J.); suzukaze@naver.com (S.-H.L.)
2 Research Center for Bioconvergence Analysis, Korea Basic Science Institute, 162 Yeongudanji-Ro, Ochang-Eup, Cheongju, Chungbuk 28119, Republic of Korea; ksryu@kbsi.re.kr (K.S.R.); keh@kbsi.re.kr (E.H.K)
3 Genome Editing Research Center, Korea Research Institute of Bioscience and Biotechnology (KRIBB), Gwahak-ro, Yuseong-gu, Daejeon 34141, Republic of Korea; jinhyuk@kribb.re.kr
4 Department of Bioinformatics, KRIBB School of Bioscience, University of Science and Technology (UST), 217 Gajung-ro, Yuseong-gu, Daejeon 34113, Republic of Korea
5 Department of Biotechnology, College of Biomedical and Health Science, Konkuk University, Chungju, Chungbuk 27478, Republic of Korea; wonhs@kku.ac.kr
6 College of Pharmacy and Department of Molecular Science and Technology, Ajou University, Suwon, Gyeonggi 16499, Republic of Korea
† These authors contributed equally to this work
* Correspondence: mdseo@ajou.ac.kr (M.-D.S.); nmrjhkim@cbnu.ac.kr (J.-H.K.)

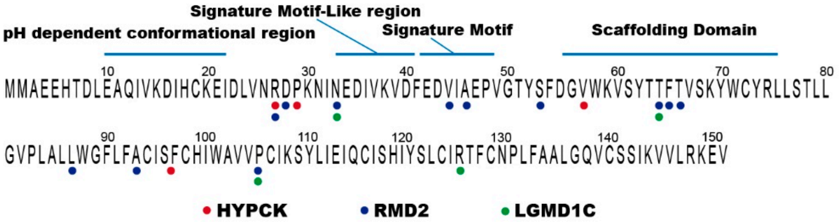

Figure S1: Disease-related amino acids of Cav3. HYPCK, HyperCKemia; RMD2, rippling muscle disease-2; LGMD1C, limb-girdle muscular dystrophy (LGMD) type 1C.

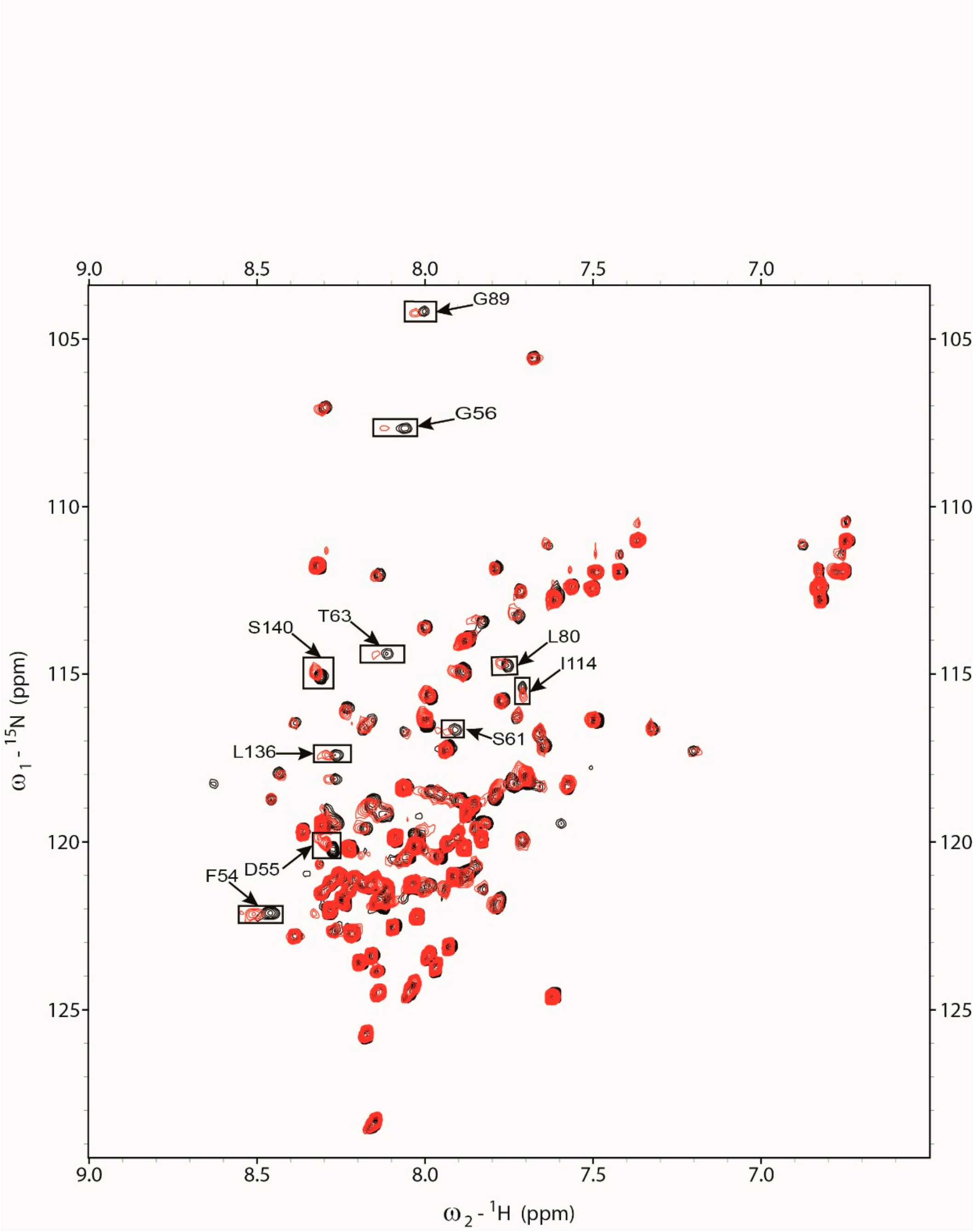

Figure S2: HSQC spectra of Cav3 in LPPG micelle with (red)/without (black) cholesterol.
